# Calcium Imaging with Super-Resolution Radial Fluctuations

**Published:** 2018-12-21

**Authors:** Yuan-Hao Lee, Shuo Zhang, Cheryl Kyles Mitchell, Ya-Ping Lin, John O’Brien

**Affiliations:** 1 Department of Ophthalmology and Visual Science, University of Texas Health Science Center at Houston, Houston, USA; 2 Department of Ophthalmology, Emory University, School of Medicine, Atlanta, USA; 3 Department of Ophthalmology, Xiangya Hospital, Central South University, Changsha, P. R. of China; 4 The MD Anderson Cancer Center UTHealth Graduate School of Biomedical Sciences, Houston, USA

**Keywords:** Calcium Signals, SRRF, Image Resolution

## Abstract

Calcium signals act as a ubiquitous secondary messenger in regulating many body functions. The detection of calcium microdomain signals is greatly facilitated by the existence of biomarker-targeted fluorescent probes. In this study, SRRF (super-resolution radial fluctuations) algorithm were used to compare the loci and the intensity of fluorescent probes before and after SRRF analysis. The implementation of SRRF algorithm was aimed for automatically resolving delicate and small calcium signals (to avoid the overlapped loci) on original images. For assessing the spatial accuracy of image intensity, immunofluorescence staining of retina cryostat slice for connexin 36 (Cx36) was microscopically imaged with or without the successive SRRF reconstruction. For characterizing the temporal association between SRRF and non-SRRF images, the changes of Cx36-GCaMP calcium indicator were recorded from transfected HeLa cells in response to the transient puffing of ionomycin. Image processing and analyses were conducted with Image J and Matlab. Through this study, SRRF reconstruction was found to confer an accurate measure for the identification of subcellular molecules, such as gap junctions. Compared with the conventional imaging, SRRF reconstruction generated better image resolution for the precise registration of individual signals. Temporally, the ratios of change in fluorescence intensity between SRRF and non-SRRF images were significantly correlated in the presence or absence of the subtraction of high background intensity. Quantitatively, the ratios of change in fluorescence intensity between SRRF and non-SRRF images with or without background subtraction were also significantly correlated. The merit of SRRF application on calcium live imaging was validated with the reporter gene system we worked on.

## 1. Introduction

Cellular calcium signals have been known as complex spatio-temporal forms that may spread as waves within cells and in between cells [1]. Imaging methods of calcium signals, hence have been developed and provide not only the temporal but also the spatial patterns of transmission [2]. Light scattering within cells, however, persists and degrades the spatial resolution of calcium signals when conventional wide-field and confocal microscopy are applied [1, 2].

**Figure 1.**
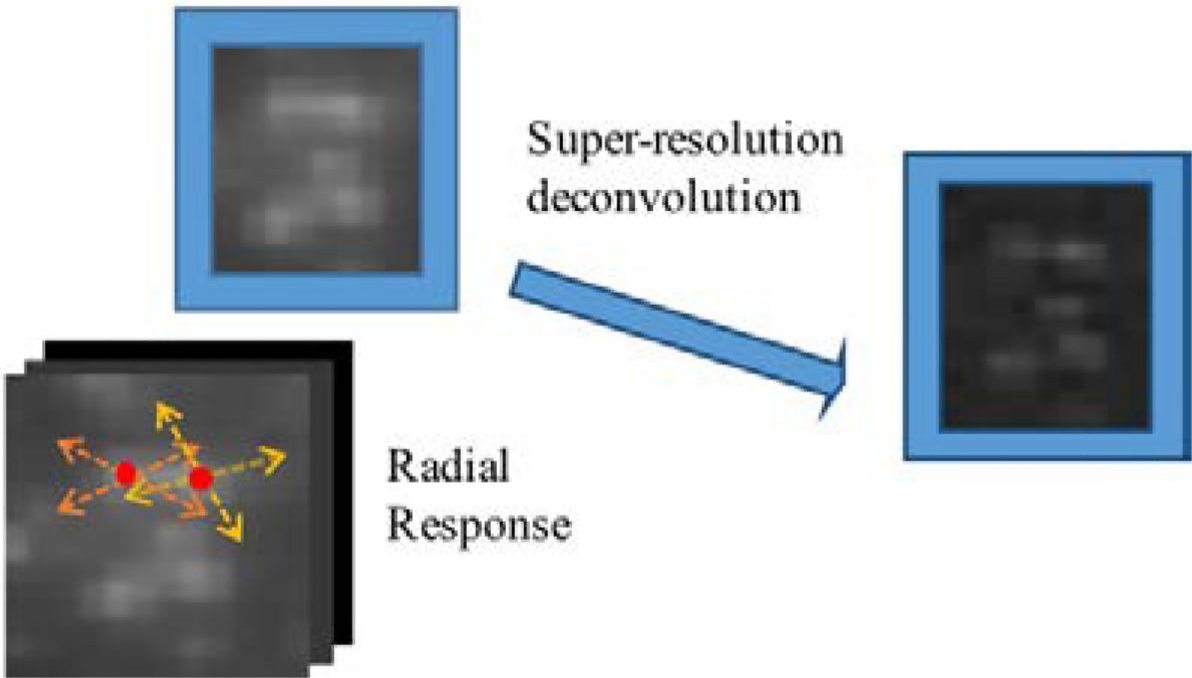
Basics of SRRF reconstruction. The radial propagation of light from fluorophores exhibited on non-SRRF image before super-resolution deconvolution, which improves the radial resolution in the spatial domain. Dotted arrows denote the radial directions from fluorophores that are represented by red round dots.

In this study, a novel analytical approach, super-resolution radial fluctuations (SRRF) algorithm was applied (Figure 1), in hopes that calcium signaling in the submicron regions can be detected. SRRF process derives a single super resolved output image from a single acquisition of multiple images. Depending on the original brightness and the correlation of fluorescence fluctuations in every pixel of sequential images, SRRF analyzes local intensity gradients across every point and magnifies each pixel into subpixels with assigned non-binary values in relation to the probability of containing a fluorophore [3].

Compared with diffraction-limited image resolution on conventional microscopy, the microscope point spread function through SRRF analysis gives rise to subpixel high resolution [3]. Studies have shown that the image quality varied depending on factors including number of frames per image, exposure time and illumination intensity, from one type of fluorophores to the other under specific imaging settings [3, 4].

## 2. Materials and Methods

### 2.1. Experimental Schemes

In order to use SRRF algorithm to facilitate the investigation of calcium signaling, the numbers of immunofluorescent labelled connexin 36 (Cx36) gap junctions in mouse retina were first spatially correlated with or without SRRF reconstruction. Secondly, the changes of Cx36-GCaMP calcium indicator expressed in HeLa cells were investigated in response to ionomycin treatment temporally. Developed by Junichi Nakai, GCaMP is created from a fusion of circularly-permuted green fluorescent protein, calmodulin and M13, a calmodulin binding site from myosin light chain kinase [5]. When calcium is bound, calmodulin undergoes a conformational change and becomes able to bind on M13. This change results in rapid de-protonation of the fluorescent protein chromophore via eliminating a water pathway and increased fluorescence [6, 7].

### 2.2. Experimental Materials

#### 2.2.1. Animal and Cell Line

The wild-type mice used in this study were C57BL6/J (stock # 000664) from The Jackson Laboratory (Sacramento, CA, United States); the mice were maintained on a daily 12 h light/ dark cycle according to the procedures approved by the Animal Welfare Committee at the University of Texas Health Science Center at Houston [8]. HeLa cells (catalog # CCL-2) were obtained from American Type Culture Collection (Rockville, MD, United States); cells were grown in complete minimum essential medium supplemented with 10% fetal bovine serum and 1% antibiotic-antimycotic [9].

#### 2.2.2. Antibodies, Plasmid DNA and Reagent

The primary and secondary antibodies used for the immunofluorescence staining of Connexin 36 were anti-Cx36 mouse IgG1 (MAB3045; EMD Millipore, Burlington, MA, United States) and Alexa Fluor 488 donkey anti-mouse IgG (A-21202; Invitrogen, Waltham, MA, United States), respectively. A plasmid expressing GCaMP3 was a gift from Loren Looger (Addgene plasmid #22692) [10]. GCaMP3 was fused to the C-terminus of mouse Cx36 to produce a Cx36-localized calcium indicator. Ionomycin dissolved in dimethyl sulfoxide was made into 5 μM with Ames medium (Fisher Scientific, Fair Lawn, New Jersey, USA).

### 2.3. Methods

#### 2.3.1. Dissection and Immunofluorescence Staining

Dark-adapted mice were anesthetized with isoflurane before cervical dislocation. The eye cups were dissected and placed in fixation buffer containing 2% N-(3-Dimethylaminopropyl)-N’-ethylcarbodiimide hydrochloride (Sigma Aldrich, Saint Louis, MO, United States) for 30 minutes at room temperature in the dark. After rinsing with PBS, fixed tissues were cryoprotected in 25% sucrose overnight at 4°C. Afterwards, tissues were embedded in O.C.T. compound and stored at −80°C. Cryosectioning of ocular tissues was performed at the slice thickness of 20 μm using a cryostat. Blocked with 10% donkey serum in PBS with 0.6% Triton X-100 at room temperature for two hours, sections then were incubated with the primary antibody overnight, followed by extensive washing with PBS and two-hour incubation with the secondary antibody-containing 0.2% Triton X-100 in PBS. Sections were preserved in Vectashield mounting medium (Vector Laboratories, Burlingame, CA, United States).

#### 2.3.2. Transfection

HeLa cells were plated on 12 mm cover glasses and transiently transfected with 2 μg of plasmid DNA per 35 mm culture dish using GenePORTER 2 transfection reagent (Genlantis, San Diego, CA, United States) upon 75% cell confluence. Microscopic live imaging was conducted 24 hours after transfection.

#### 2.3.3. Imaging

Immunofluorescently labelled retina sections were imaged with an ANDOR iXon Life 897 EMCCD camera (Oxford instruments, Abingdon, United Kingdom) on an Olympus BX51WI fluorescence microscope with or without SRRF reconstruction. The loci of Cx36 were identified by the green fluorescence (λ_Ex_: 450–490 nm, λ_Em_: 500–550 nm) through a water immersion objective, Olympus LUMPlanFL lens 40x/0.80 N.A. Live cell imaging was conducted with Cx36-GCaMP-transfected HeLa cells superfused with 37°C pre-warmed and oxygenated Ames medium perfused by a peristaltic pump (Model P720, Instech Laboratories, Plymouth Meeting, PA, United States). The GCaMP loci were imaged with a doubler in addition to Olympus LUMPlanFL lens 40x/0.80 N.A. water immersion objective through the bandpass filters (λ_Ex_: 490–510 nm, λ_Em_: 520–550 nm) for quantitatively measuring the effect of transient ionomycin puffing on GCaMP fluorescence.

#### 2.3.4. Transient Ionomycin Puffing

Puffing of ionomycin was performed with patch pipette (Borosilicate Glass With Filament # BF120–94-10, Sutter Instrument, Hofheim, Hesse, Germany) that were loaded with 10 μl of ionomycin solution and with the pipette tip located adjacent to cells within the field of view. Ionomycin solution was puffed into the perfusion chamber with the settings of the injection pressure at 0.2 psi and the injection time at two seconds using a Pico-Injector (Model PLI-100, Harvard Apparatus, Holliston, MA, United States).

#### 2.3.5. Image Processing

The 16-bit images of retina slides were processed through Image J 1.52e (National Institutes of Health, Bethesda, MD, United States) and assessed with Matlab R2018a (MathWorks, Natick, MA, United States) whereas live cell images were analysed with Matlab R2018a. SRRF image resizing was performed prior to image analysis. For evaluating the intensity and spatial relations of Cx36 between rescaled SRRF and non-SRRF images, point-by-point local maxima recognition was processed through setting optimal image noise tolerances. On the other hand, the loci and expression levels of Cx36-GCaMP in Hela cells were found by creating a ROI (regions of interest) map via maximum intensity projection (MIP) for the time phase direction as well as pixel-wise matching-up of row and column maxima in the spatial domain. The ratio of change in fluorescence intensity (ΔF/F_0_) over approximately 30 seconds (for 30 time points) was used as an indicator of calcium signals.

#### 2.3.6. Statistical Analyses

The linear relationship of image intensity and the temporal association between rescaled SRRF and non-SRRF images were analyzed with Pearson product-moment correlation and Spearman rank correlation, respectively. Statistical analyses were performed using Excel; a value of p < 0.05 was statistically significant.

## 3. Results

### 3.1. Evaluation of Intensity and Spatial Relations

First, the spatial relation of Cx36 loci were assessed between SRRF and non-SRRF images. Then, the intensity was compared. Imaging settings are listed in Table 1.

**Figure 2.**
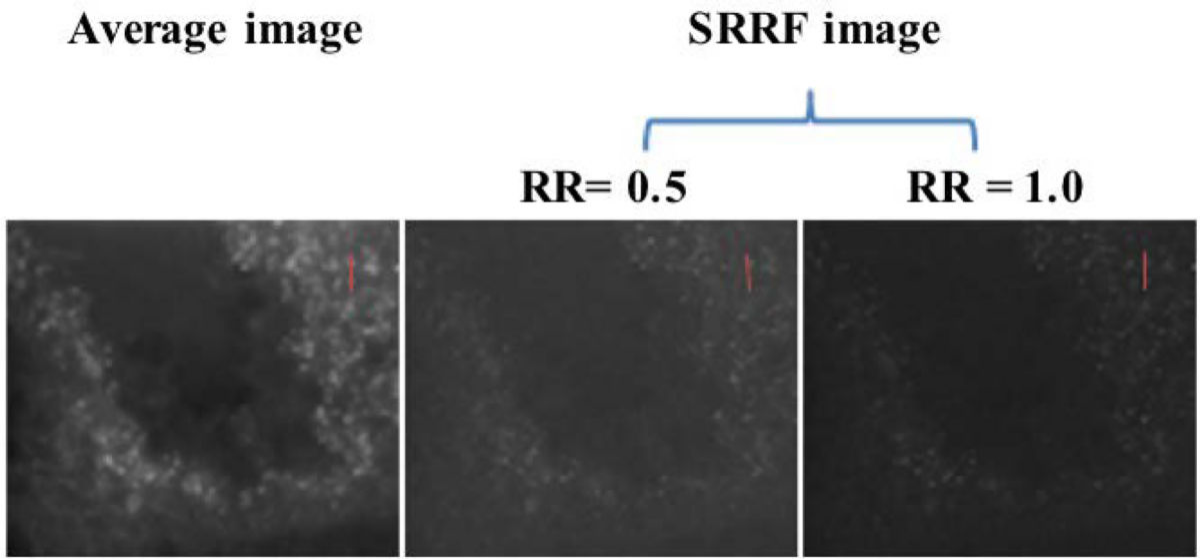
Images of fluorescent loci acquired with or without SRRF reconstruction simultaneously at different imaging conditions: Each image was generated from 30 frames. Catmull-Rom was used as the interpolation method for image reconstruction. Abbreviation: RR stands for ring radius. Note: The average image was acquired using the same number of frames as the SRRF image. Note: Red lines indicate linear locations sampled for the analysis of intensity profiles.

**Figure 3.**
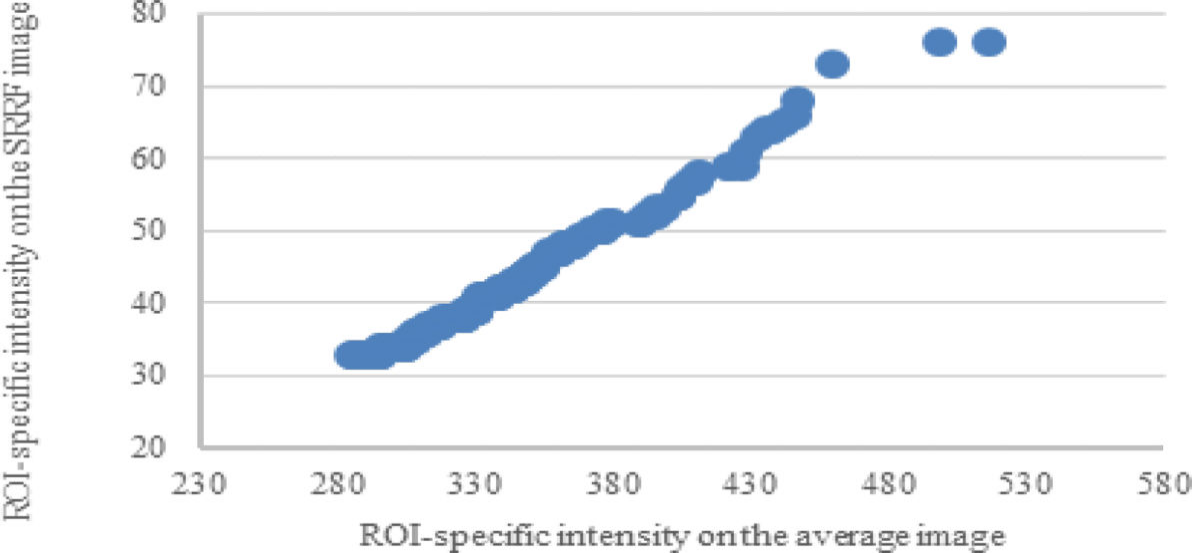
Loci intensity correlation of average and SRRF image intensity: The strength of correlation between the average image and the SRRF (ring radius = 0.5) was described by Pearson’s correlation coefficient (γ = 0.9943) and p value (p < 0.0001).

Experimental Results: The local maxima estimated through ImageJ indicate a higher noise level in the average image than in the SRRF image (Figure 2). The noise tolerance limits for processing the average and SRRF images are 30, 10 (ring radius, 0.5) and 11 (ring radius, 1.0), respectively. By increasing the ring radius of SRRF reconstruction, the noise level of reconstructed image was also increased. Nevertheless, the loci of Cx36, identified as local maxima, on average image have 89% spatial similarity with those on the SRRF image. Statistically, the loci intensity of SRRF image is significantly correlated with that of average image (Figure 3).

**Figure 4.**
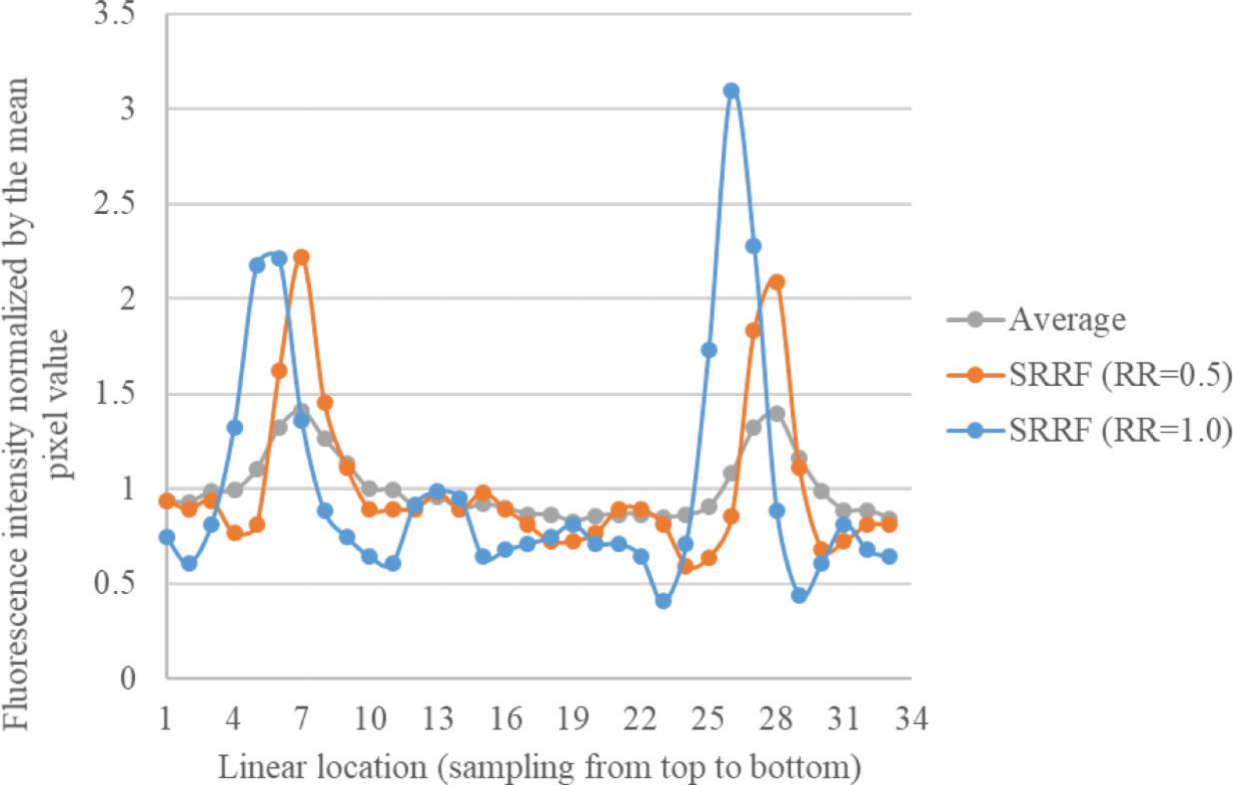
Intensity profiles of two fluorophores analyzed with different images: The three profiles delineate the normalized pixel intensity of linear locations described in Figure 2.

Tissue movement during live imaging can make loci recognition difficult. Fortunately, SRRF images revealed a higher resolution compared with the average image (Figure 4). This result indicates that SRRF images can be used as a mask for localizing local maxima.

### 3.2. Evaluation of Temporal Association

**Figure 5.**
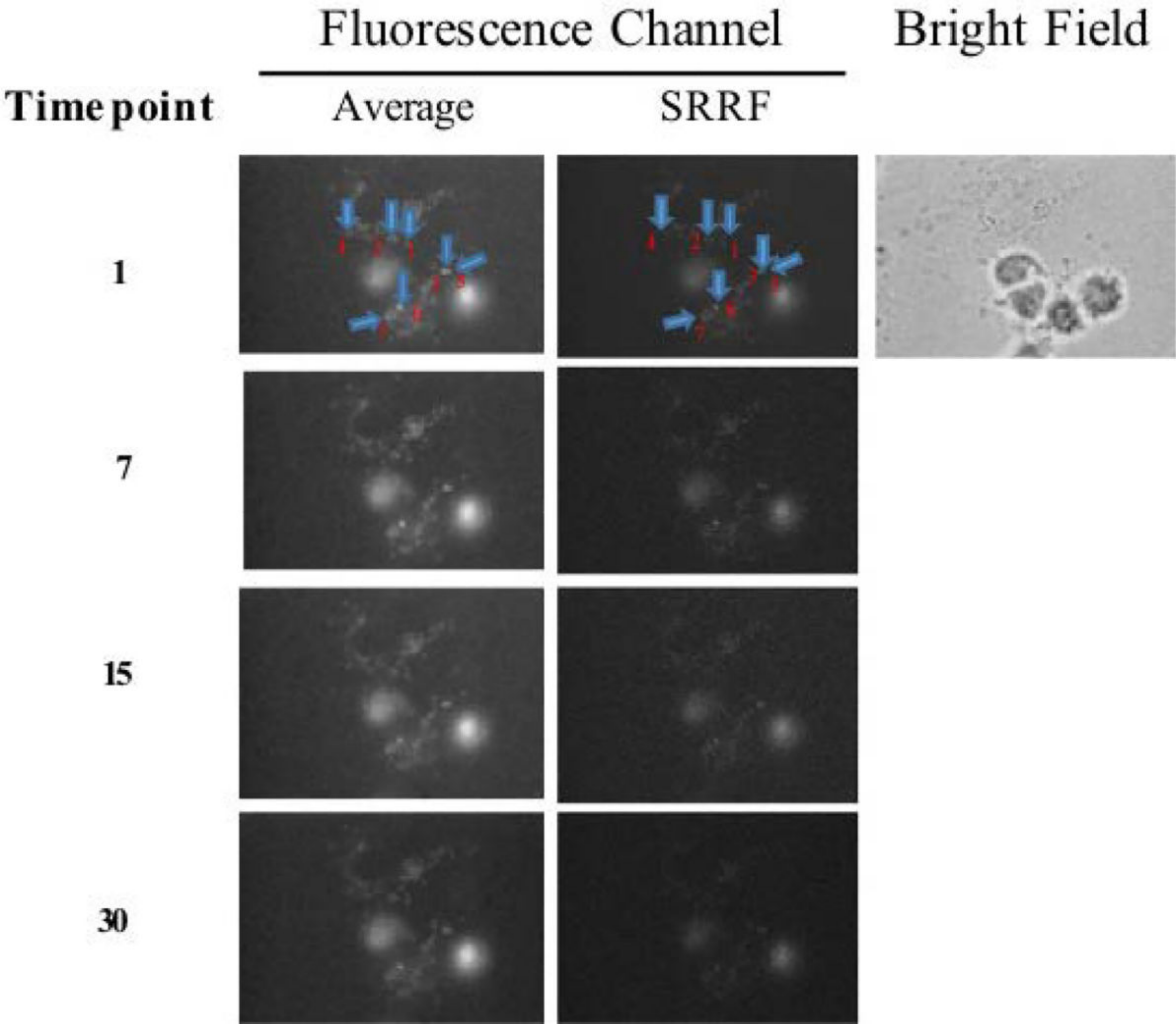
Live imaging of calcium signals through a time course: The acquisition of average and SRRF images adopted an exposure time of 10 ms (30 frames per image) with an electron-multiplying gain of 200. Arrows and numbers denote seven regions of interest at the gap junctions for the analysis of temporal changes of fluorescence intensity. Note: The brightness and contrast of SRRF images were increased by 20% and 40%, respectively, for the better visualization of GCaMP spots.

To evaluate the capability of SRRF algorithm in facilitating conventional live imaging, fluorescence images of Cx36-GCaMP were acquired with or without SRRF reconstruction separately, and the temporal association were compared in correspondence to ionomycin puffing (Figure 5). The imaging settings are described in the caption of Figure 5.

**Figure 6.**
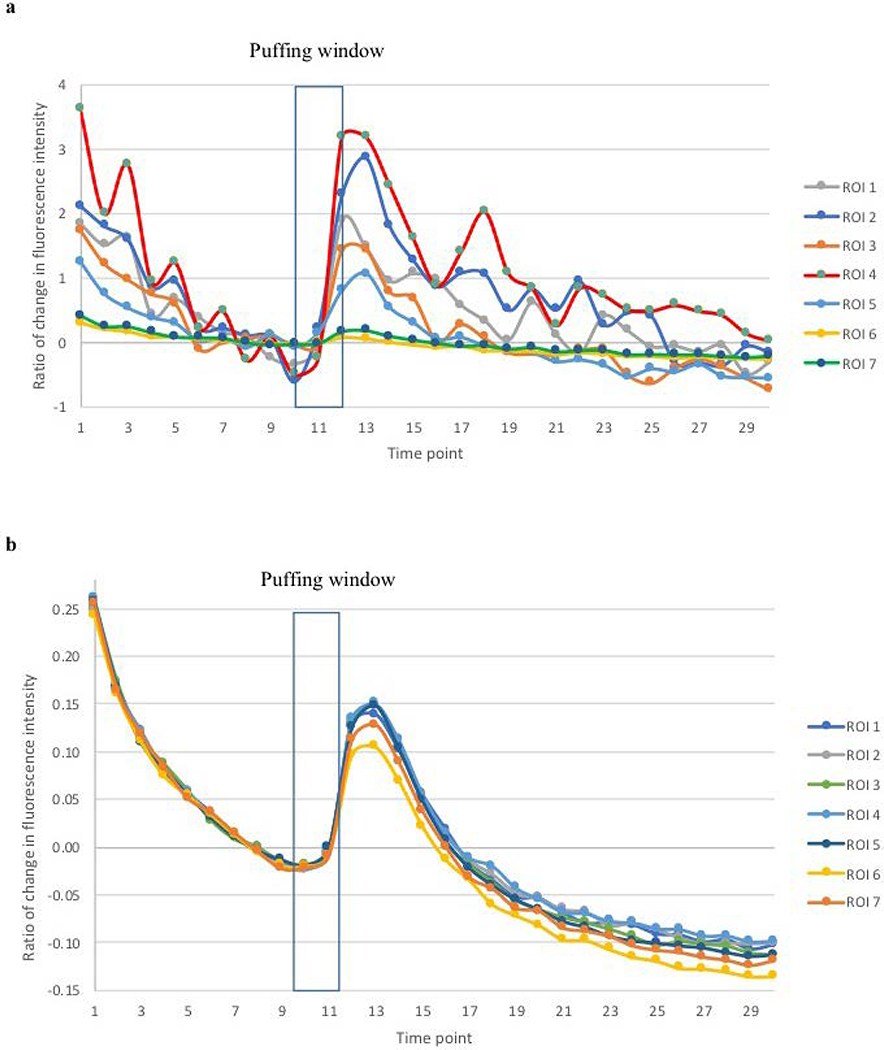
The temporal changes of fluorescence intensity in average images: Profiles in panels a & b represent image analyses with or without background subtraction. Ionomycin solution was puffed upon the completion of the 10^th^ image acquisition for two seconds. The intensity changes were expressed as ΔF/F_0_; each of the regions of interest (ROI, size: 15-by-15 pixels) corresponds to the labeled foci in Figure 5.

**Figure 7.**
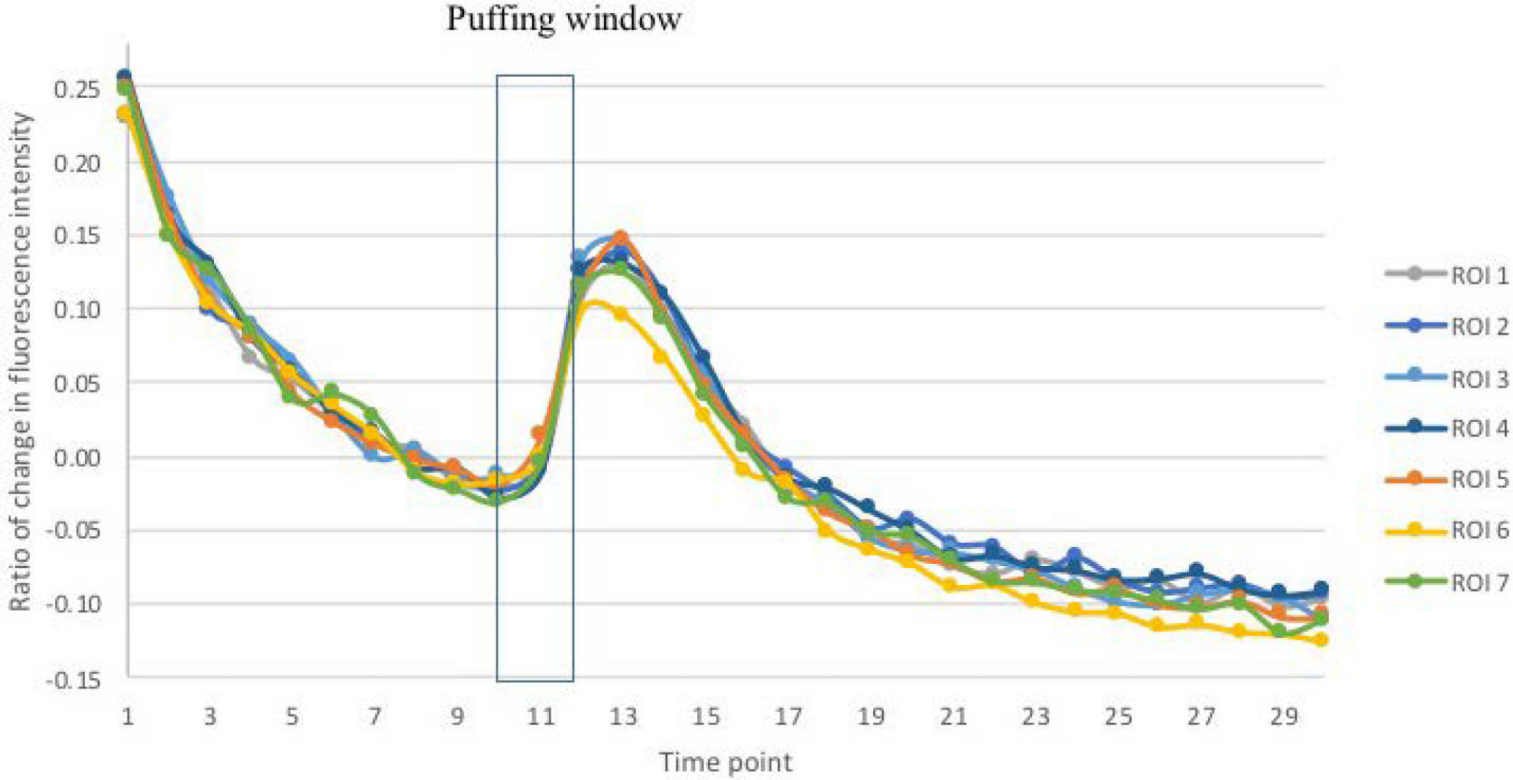
The temporal changes of fluorescence intensity in SRRF images without background subtraction: Ionomycin solution was puffed during image acquisition as denoted in Figure 6. Seven regions of interest (ROIs 1~7) were corresponding to the labeled foci found in Figure 5.

Experimental Results: The temporal changes of GCaMP fluorescence intensity are depicted in Figures 6 and 7. In response to ionomycin puffing, fluorescence signals from GCaMP ROIs increased transiently, as did background signals (data not shown). Statistically, all values of ΔF/F_0_ measured from average images with background subtraction were significantly correlated with those measured from average images without background subtraction (Pearson’s correlation coefficient, γ (208) = 0.6828; p < 0.0001) as well as those measured from SRRF images (γ (208) = 0.6790; p < 0.0001). Nevertheless, the values of ΔF/F0 measured from SRRF images are most correlated with those measured from average images without background subtraction (γ (208) = 0.9983; p < 0.0001).

From the time perspective, SRRF reconstruction reflected real-time changes in fluorescence intensity, which is significantly correlated with those depicted by live average images (Table 2).

## 4. Discussion

Random and radial fluctuations are readily captured by optical detectors as noise or undesired signals [11]. Usually, the way to reduce photon noise is to capture more signals [12]. But this can increase phototoxicity to live tissues. In contrast, the convergence of fluorophores’ illuminance through SRRF reconstruction acts like reducing the field of view of the detector so as to view the source of interest without receiving background signals.

In this study, SRRF algorithm was first applied for determining the intensity of Cx36 fluorophores at different ring radiuses. By comparing with the intensity detected from the non-SRRF image, the radiality transform profile at the ring radius of 0.5 was found to exhibit a greater degree of similarity in location and fluorescence intensity. In theory, the optimal choice of the ring radius relates to the level of radial symmetry of the imaged fluorophores [4]. An image generated from larger numbers of frames tends to result in less variation for the reconstruction of the point spread functions using lower values of the ring radius. Here, the ring radius was kept at 0.5 for applying SRRF algorithm to calcium imaging.

In our experiments, the ΔF/F_0_ profile measured in SRRF images corresponded temporally to that measured from average images. Although there is a significant correlation between the temporal changes in the amplitude of responses measured from SRRF image and average images, the ΔF/F_0_ measured from average images that were processed with background subtraction was affected by the high level of background intensity. This background was correlated with GCaMP responses and likely representing fluorescence signals scattered from GCaMP-containing structures. Consequently, subtraction of the background may depress measurement of valid fluorescent responses from GCaMP ROIs. Not surprisingly, the correlation in amplitude of fluorescent responses was higher between SRRF and average images without background subtraction than with background subtraction.

## 5. Conclusion

This study demonstrated the feasibility of implementing the SRRF algorithm for live calcium imaging via the application of image analysis software. A significantly high spatial correlation between non-SRRF and SRRF images was first assessed with the graphic software, ImageJ, which served as a standard method for validating the image processing and assessment methods developed in Matlab. Through the application two-dimensional and one-dimensional MIP on a stack of live SRRF images and the resultant MIP image, respectively, the locations of local maxima loci on a set of SRRF images were identified through the backprojection and matching-up of row and column maxima. By comparing the ΔF/F_0_ profiles calculated from the pixel values of identified local maxima loci, significantly strong temporal associations between non-SRRF and SRRF images were verified. The characterized results support the implementation of SRRF algorithm for live calcium imaging.

## Figures and Tables

**Table 1.** Imaging settings.

Parameter (unit)	Value
Exposure (ms)	10
Andor	
Electron-multiplying gain (arb.)	74
SRRF status	
No. of frames per time point (arb.)	100
Radiality magnification (arb.)	4

**Table 2.** Spearman rank correlation between SRRF and non-SRRF (or average) images.

Region of interest	Average image with background subtraction		Average image without background subtraction	
	ρ	p-value	ρ	p-value
1	0.796	< 0.0001	0.996	< 0.0001
2	0.747	< 0.0001	0.994	< 0.0001
3	0.942	< 0.0001	0.992	< 0.0001
4	0.623	0.0002	0.995	< 0.0001
5	0.968	< 0.0001	0.995	< 0.0001
6	0.977	< 0.0001	0.997	< 0.0001
7	0.990	< 0.0001	0.995	< 0.0001
